# Therapeutic hypothermia: the rationale

**DOI:** 10.1186/cc11260

**Published:** 2012-06-07

**Authors:** Erich Schmutzhard, Marlene Fischer, Anelia Dietmann, Gregor Brössner

**Affiliations:** 1 Department of Neurology, Neurocritical Care Unit, Medical University Innsbruck, Austria

## 

For almost a century, therapeutic hypothermia - or as it was termed in the early days: hibernation - has been discussed as a potential neuroprotective measure, in particular in patients suffering from severe intracranial disease leading to impairment of consciousness, associated with fever [1-3].

In a wide range of diseases, secondary damage to the brain or other organs follows the initial impact and may be responsible for aggravation of disease condition or clinical state, in particular neurological morbidity and/or mortality [4-11]. Therapeutic hypothermia, recently renamed targeted temperature management, including prophylactic normothermia, has been used to improve this secondary impact onto brain and other organ tissue. This holds true, in particular, for neurological and neurosurgical intensive care patients since secondary brain and nervous tissue injury may preclude a potentially benign course of disease. The mechanisms of action of hypothermia are complex, not yet fully understood. Therapeutic hypothermia/targeted temperature management aims to attenuate a cascade of secondary injury mechanisms, which is started immediately after the initial event (primary injury) and may last for hours and even days [4,6,12]. The majority of research has focused, so far, on these secondary injury processes being destructive to brain and nervous tissue. It may be expected that any such protective effect can be replicated in other organs and tissues during therapeutic hypothermia/targeted temperature management. A wide range of side effects may negate and counteract its positive initial effect; this implies side effects of hypothermia *per se *and side effects of rewarming or inconstant maintenance of temperature levels [13-17].

This abstract limits itself to potential pathophysiological mechanisms of actions, the risks of any such mechanism and side effects derived from them [4,5,10,12,16-18].

The protective effect of hypothermia may be explained by several pathways. A *decreased metabolism *with *less oxygen *and *energy consumption *and *carbon dioxide production *may prevent secondary injury when oxygen supply is interrupted or, at least, impaired. However, it needs to be stressed that the reduction in metabolic rate, as seen in hypothermia, requires adjustment in ventilator setup, insulin infusion rate, correct interpretation of electrolytes, in particular low phosphate, magnesium and potassium levels. Of particular interest are the rebound phenomena during rewarming or when, involuntarily, the temperature cannot be maintained at the targeted low level.

Following ischemia, hypoxia or direct trauma apoptotic processes may be initiated in brain tissue and neuronal cells may even become *necrotic*. In these earliest stages these pathways may be blocked by hypothermia. However, little is known about the time frame and best window of opportunity to use therapeutic hypothermia to prevent initiation of apoptotic/necrotic processes.

Any type of neuronal injury may provoke the *neuro-excitatory cascade*, starting off with excessive *calcium influx, glutamate receptor activation, neuronal hyperexcitability*, eventually leading to cell death even after reperfusion and normalization of glutamate levels. It has been suggested that therapeutic hypothermia may reduce cellular/neuronal damage following this neuro-excitatory cascade.

It has been accepted that the *release of free radicals *may be deleterious to both neuronal cells and the brain's defense mechanisms alike. Whether the direct impact or the ischemia reperfusion injury is overwhelmingly responsible for the release/increase of free radicals oxidizing and damaging neuronal cell components is both still a matter of discussion and of limited interest when therapeutic hypothermia comes into play.

Hyperexcitability, cellular hyperactivity, mitochondrial dysfunction, ion-pump failure and reduction in cellular membrane integrity may lead to intracellular and, consequently, also *intercellular/extracellular acidosis*.

Early initiation of hypothermia may improve this full spectrum of cellular failure, improve brain glucose and energy metabolism and reduce lactate accumulation; with this, intracellular and intercellular acidosis will improve and eventually metabolic recovery be enhanced [4,19-21].

Any type of brain injury is capable of *disrupting the blood brain barrier *leading to enhanced vascular permeability, brain edema, vascular permeability and perivascular hemorrhage. *Brain edema*, both after ischemia/hypoxia and traumatic injury peaks after 24 to 72 hours (sometimes reaching its highest peak even after this period of time) - thus opening widely the therapeutic window - allowing for therapeutic hypothermia to reduce brain edema via stabilizing the disrupted blood-brain barrier and vascular permeability. After brain injury *proinflammatory mediators are released*, leucocytes cross the - already impaired - blood-brain barrier leading to an *accumulation of inflammatory cells in the br*ain. This inflammatory response starts within 1 hour after injury and may persist for up to 5 days, a fact which also suggests a widely open therapeutic window for intervention. Hypothermia has been shown to reduce ischemia induced inflammatory and immune reactions [4,19-22].

In healthy persons, brain temperature is around 0.5 to 1°C higher than core body temperature. In any type of brain injury, in particular, in patients with fever or hyperpyrexia respectively, injured areas may be definitely hotter (up to 2°C post injury), most probably due to transitory cellular hyperactivity. Local brain edema might lead to cerebral thermopooling adding to *hyperthermia-related neuronal cellular injury *[4,16,18-21].

Cooling below 35°C has been shown to affect coagulation, it depends on the initial type of brain injury whether a procoagulatory effect or an anticoagulatory effect is believed to be neuroprotective in an individual case.

Targeted temperature management may influence the secretion of vasoconstricting substances (for example, endothelin) or vasodilating substances (for example, prostaglandins). Their balance is essential to maintain homeostasis. Ischemic or traumatic conditions may increase vasoconstricting substances thus leading to reduced cerebral blood flow. Whether hypothermia is capable of regulating/improving cerebral perfusion is still a matter of investigation, pending the influence of cerebral autoregulation and the quantity of secreted vasoactive mediators in brain-injured patients with cerebral ischemia or any other type of injury [10].

Whether epileptic activity, in particular, subtle nonconvulsive status epilepticus, accepted to indicate severe brain damage, can be positively influenced by therapeutic hypothermia still needs further research. However, it is accepted that a subtle nonconvulsive status epilepticus occurring in the acute phase of brain injury is - *per se *- adding to neuronal destruction [10,16].

While many pathophysiological processes and cascades may be influenced by targeted temperature management/therapeutic hypothermia and/or even prevention of fever through prophylactic normothermia, it is unclear whether in all types of severely brain-injured patients (for whatever reason) the benefits of this therapeutic hypothermia always outweigh its risks. It is now fully accepted and of a high level of evidenced medicine that in cerebral hypoxia (in a patient with cardiac arrest due to a shockable arrhythmia) as well as asphyxial encephalopathy a 24-hour therapeutic hypothermia (33 to 34°C), irrespective of the type of cooling, improves neurological outcome; that is, morbidity but also mortality [7,10]. Whether therapeutic hypothermia/targeted temperature management or prophylactic normothermia may improve outcome in other diseases, as discussed in this meeting, is still not clear. It needs to be stressed that even such seemingly similar diseases as global hypoxia (in cardiac arrest due to a shockable arrhythmia), asphyxial encephalopathy and ischemic stroke have so few pathophysiologic cascades in common. Therefore, they may not be treated all alike, in particular, with respect to type, duration, speed and depth of hypothermia as well as rewarming management [23]. It has already been demonstrated that in hypoxic encephalopathy hypothermia for 24 hours may be sufficient. However, disease entities such as ischemic stroke, hemorrhagic stroke with formation of peri-hematomal edema, traumatic brain injuries with prolonged secondary insult or the wide range of neuronal injuries after subarachnoid hemorrhage may present even more complex pathophysiologic mechanisms. Moreover, different pathologies such as encephalitis and bacterial meningitis or even spinal cord injury may all require a targeted and personalized approach to this adjunctive therapy. In some cases, prolonged hypothermia may be equally necessary as in other cases mild hypothermia or even only prophylactic normothermia may suffice.

It may be stated beyond doubt that the neuroprotective effect of moderate hypothermia (33 to 34°C) has been shown in cerebral hypoxia and asphyxial encephalopathy. However, different neurocritical care disease entities as discussed above have different mechanisms of primary insults as well as the mechanisms and cascades of secondary brain injury and therefore require a different therapeutic approach in respect of temperature management.

Any type of therapeutic measure, still being the subject of research, must never harm the patient. Hypothermia-induced neurological signs and symptoms must never be misinterpreted and as a matter of course the diagnosis of brain death can never be confirmed under hypothermic conditions [24].
